# Histone Marks-Dependent Effect on Alternative Splicing: New Perspectives for Targeted Splicing Modulation in Cancer?

**DOI:** 10.3390/ijms23158304

**Published:** 2022-07-27

**Authors:** Carol Imbriano, Silvia Belluti

**Affiliations:** Department of Life Sciences, University of Modena and Reggio Emilia, Via Campi 213/D, 41125 Modena, Italy

**Keywords:** alternative splicing, histone post-translational modifications, histone-code, cancer transcript variants, epigenome editing

## Abstract

Alternative splicing (AS) is a tightly regulated mechanism that generates the complex human proteome from a small number of genes. *Cis*-regulatory RNA motifs in exons and introns control AS, recruiting positive and negative *trans*-acting splicing regulators. At a higher level, chromatin affects splicing events. Growing evidence indicates that the popular histone code hypothesis can be extended to RNA-level processes, such as AS. In addition to nucleosome positioning, which can generate transcriptional barriers to shape the final splicing outcome, histone post-translational modifications can contribute to the detailed regulation of single exon inclusion/exclusion. A histone-based system can identify alternatively spliced chromatin stretches, affecting RNAPII elongation locally or recruiting splicing components via adaptor complexes. In tumor cells, several mechanisms trigger misregulated AS events and produce cancer-associated transcripts. On a genome-wide level, aberrant AS can be the consequence of dysfunctional epigenetic splicing code, including altered enrichment in histone post-translational modifications. This review describes the main findings related to the effect of histone modifications and variants on splicing outcome and how a dysfunctional epigenetic splicing code triggers aberrant AS in cancer. In addition, it highlights recent advances in programmable DNA-targeting technologies and their possible application for AS targeted epigenetic modulation.

## 1. Introduction

Alternative splicing (AS) is a versatile, tightly regulated mechanism that explains how the complexity of the human proteome is formed from a small number of genes. This is also a critical step in gene expression regulation. AS events convert a pre-mRNA molecule into several mature mRNAs that can be translated into different proteins, thereby expanding proteome complexity [1,2]. In addition, AS events occurring in untranslated regions (UTRs) of mRNA, which do not affect the polypeptide sequence, can modulate mRNA behavior ([3,4], reviewed in [5]). Hence, alternatively spliced transcripts can be differentially regulated in terms of cellular localization, stability, translational activity and, when translated into proteins, can result in isoforms with distinct structure and function.

Although AS occurs in more than 90% of multiexonic human genes, according to high-throughput investigations of the human transcriptome [6,7,8], the mechanisms behind cell type- or stage-specific selection of appropriate exons is largely unresolved. AS is frequently tightly regulated by cell type, developmental stage, or both, and dysregulation of AS is related to a variety of genetic and hereditary disorders. Moreover, aberrant or misregulated RNA splicing events contribute to cancer cell phenotypes via influencing the cell genome, epigenome, transcriptome, and proteome, either directly or indirectly [9,10,11]. AS variant proteins can differ in stability and biological functions, due to the loss or gain of functional domains (e.g., DNA-binding sites and active sites of enzymes), changes in subcellular localization, or the modification of protein interactions with substrates and other proteins. In addition to the clearly expected consequences of dysregulated AS of transcriptional regulators, such as transcription factors [11,12], aberrant splicing events can alter genome stability and chromatin structure. For example, the proper splicing of proteins involved in mitotic progression and chromosome segregation are fundamental for the maintenance of genome stability [13,14]. Moreover, the compromised function of the spliceosome, one of the cancer hallmarks, has clear effects on the DNA-damage response and genome stability [15].

AS is controlled at the most basic level by the presence of *cis*-regulatory RNA motifs in both exons and introns. These RNA motifs are responsible for recruiting positive and negative *trans*-acting splicing regulators, usually RNA-binding proteins that will either favor or block the inclusion of the regulated exon in the pre-mRNA [16]. At a higher and more integrated level, it has been demonstrated that chromatin has an effect on the outcome of splicing [17,18]. This is a rapidly evolving field of study that has greatly benefited from recent improvements in genome-wide epigenetic analyses. These strategies have identified novel mechanisms regulating AS and expanded our understanding of this process.

The splicing and chromatin codes have historically been thought to operate at two distinct levels: the chromatin code works at the DNA level, while the splicing code operates at the RNA level. However, a growing body of evidence suggests that epigenetic mechanisms, such as DNA methylation, histone modifications, histone variants, and noncoding RNAs (ncRNAs), are important for RNA processing, through the dynamic regulation of specific chromatin regions. This means that the popular histone code hypothesis [19], stating that the recognition of specific combinations of histone marks by protein complexes leads to different downstream events, can now be extended to RNA-level processes.

In this paper, we review the data supporting the effect of histone modifications and histone variants on splicing outcome and how aberrant AS in cancer can be the consequence of a dysfunctional epigenetic splicing code. The potentiality of histone post-translational modifications (PTMs) as a tool to modulate alternative splicing is also discussed.

## 2. Definition of Exons at the Chromatin Level

The packing of eukaryotic nuclear DNA into nucleosomes, which contain 147 bps DNA wrapped around a histone octamer, restricts accessibility and, consequently, interferes with biological processes that use DNA as a template, such as transcription. Along with nucleosome placement, histone modification patterns denote functional genomic areas and have an effect on the recruitment of *trans*-acting factors [20]. Recently, it has been demonstrated that chromatin characteristics influence processes that do not directly involve DNA but occur concurrently with transcription, such as pre-mRNA splicing [18]. The genome-wide distribution of nucleosomes around exons in *Drosophila melanogaster*, *Caenorhabditis elegans,* and *Medaka* (*Oryzias latipes*) early embryonic cells has revealed their nonrandom distribution, with a specific positioning at the intron/exon junction, generating transcriptional barriers that can shape the final splicing outcome [20,21,22]. This supported the assumption that a well-positioned nucleosome can slow down RNA polymerase II (RNAPII) and increase the recruitment of splicing factors to the pre-mRNA. In mammalian cells also, RNAPII binding is higher on exons than introns [20,23,24], and delaying RNAPII at exons enables more time for the splicing machinery to detect and define exons. Nucleosomes are supposed to mark exons, to assist the splicing machinery in selecting short exons within long intronic tracts, and the ratio of nucleosome occupancy within exons well correlates with the levels of exon inclusion [25,26]. Despite the evidence of a connection between nucleosome positioning, RNAPII elongation speed, and splicing events, the abundance of nucleosomes in exons is also observed in nontranscribed genes, implying that exon nucleosome marking can be independent of transcription [21]. 

Furthermore, exon-specific nucleosomes are rich in certain histone PTMs, which have been demonstrated to influence the final splicing decision [20,27,28,29]. ChIP-seq data for 38 histone PTMs revealed a significant enrichment in H3K36me3, demonstrating that exon/intron boundaries are defined by transitions between areas enriched in and depleted of H3K36me3 [18]. More recently, Agirre at al. identified 11 chromatin modifications that differentially mark AS exons in a highly localized and combinatorial way [30]. The inclusion levels of selected exons well correlate with changes in the enrichment of the histone marks in different cell types, further demonstrating a role for chromatin in the recruitment of the splicing machinery to pre-mRNAs. Thus far, the kinetic coupling [17] and the chromatin-splicing adaptor systems [28,30,31] are the two models that functionally connect chromatin to splicing (reviewed in [18]).

## 3. Models for Coupling of Epigenetics, Transcription, and Alternative Splicing

Pre-mRNA splicing can occur during its transcription and happens within 5–10 min after synthesis, according to a recent analysis of large human genes [32]. For co-transcriptional splicing processes, introns are deleted while the nascent transcript is still associated with the DNA through RNAPII. 

**Figure 1:**
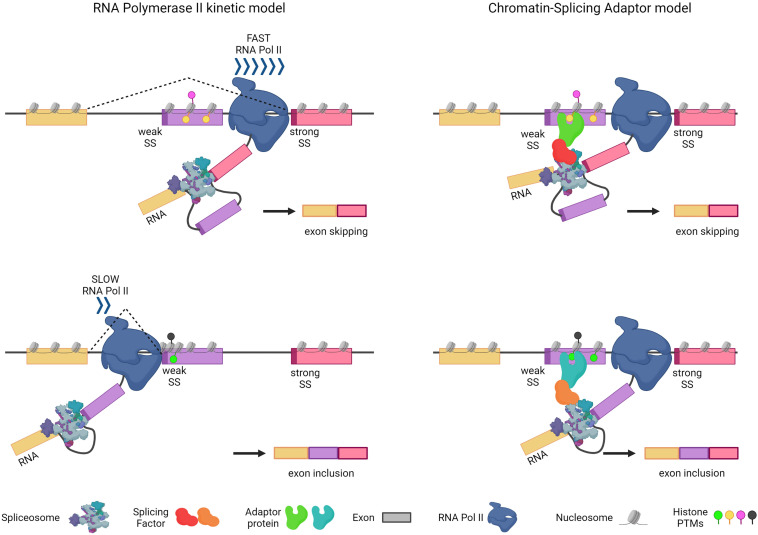
Two non-exclusive models describe how transcription and alternative splicing are functionally coupled. Left: In the context of the Kinetic Coupling Model, the rate of transcriptional elongation, influenced by different histone PTMs, affects AS outcome. At high RNA polymerase II (RNA Pol II) elongation rates, there is a brief temporal window in which weak (purple) and strong (pink) splice sites (SS) compete for the recruitment of the splicing machinery, resulting in the skipping of the weaker exon (purple rectangle). On the contrary, exon inclusion is favored by RNA Pol II slowing down or pausing, which facilitates the recognition of the weak SS before the strong SS is synthesized. Right: According to the Chromatin-Adaptor Model, histone-PTMs changes along the gene (marked in different colors) determine the recruitment of specific adaptor proteins, which, in turn, recruit splicing factors and the transcription machinery, hence influencing AS decisions. Created with BioRender.com.

The “kinetic coupling model” states that transcription elongation and splicing compete kinetically. Due to the fact that alternative exons generally have inefficient splicing signals that require additional time to be recognized by the splicing machinery, increased RNAPII elongation speed results in a greater skipping of these exons, implying a physiological role for the coupling between transcription kinetics and AS regulation [17,33]. Spliceosome machinery and splicing factors recognize splicing sites and regulatory regions on RNA as they arise. Additionally, RNAPII is connected with a large number of splicing factors via its C-terminal domain. Reversible histone PTMs are a way for rapidly modulating the structure of local chromatin during transcription. Sequences that generate transcriptional pauses, as well as chromatin compaction and the presence of RNAPII-blocking proteins, enhance the time window for weak splicing sites (SS) to be detected. Conversely, a high elongation rate increases the likelihood that strong sites are detected, as opposed to weak ones [34] (Figure 1). 

The second model that links chromatin and AS is the “chromatin-splicing adaptor system”. Chromatin remodeling proteins that recognize and bind distinct histone marks can target splicing factors to the transcription site during co-transcriptional splicing, accomplishing two objectives. To begin, core spliceosome components are recruited to enable quick and accurate intron removal. Second, chromatin remodeling proteins attract splicing regulators, which have a direct effect on the inclusion or exclusion of alternative exons [18]. Therefore, according to the recruitment model, chromatin modifications recruit chromatin-binding proteins that can modulate the presence of splicing factors to the pre-mRNA (Figure 1). In addition, it is important to highlight that kinetic coupling and chromatin-splicing adaptor systems are not mutually exclusive.

## 4. From Specific Chromatin Markers to an Epigenetic Splicing Code 

These findings clearly suggest that chromatin signatures play a critical role in regulating RNA splicing and provide important clues for elucidating the regulatory mechanism behind AS. 

### 4.1. Histone Variants 

Histone variants can take the place of canonical histones, leading to differences in epigenome function by participating in the regulation of nuclear processes, including AS.

Linker histones were shown to interact with RNAPII and a variety of splicing factors. Additionally, H1 limits nucleosome sliding, allowing the core histones to maintain their position relative to exons. There is evidence that distinct linker histone variants have distinct roles. H1.5 accumulates in differentiated human lung fibroblasts but not in embryonic stem cells in their undifferentiated state. ChIP-seq investigation of the linker histone variant H1.5 in human lung fibroblast IMR90 cells and RNA-seq analysis of wild-type and H1.5-deficient IMR90 cells revealed that H1.5 binds nearby short exons, contributes to their inclusion, and improves RNAPII pausing, increasing the likelihood that an exon would be detected by the splicing machinery [35].

The occupancy of the ubiquitous histone H2A variant H2A.Z has been demonstrated to promote co-transcriptional splicing of suboptimal introns in *Schizosaccharomyces pombe* and in *Saccharomyces cerevisiae* [36,37]. In addition, the mammalian-specific short H2A histone variant hH2A.B (originally designated H2A.Bbd) and its murine homolog mH2A.B.3, which are encoded by three X chromosome genes and are enriched in active regions of the genome mainly in the testis and brain, showed a distinct pattern in nucleosome positioning around intron–exon junctions. Genome-wide localization of nucleosomes containing H2A.B/H2A.B.3 coupled to transcriptomic and proteomic analyses [38,39] indicated that this histone variant might be involved in the regulation of transcriptional elongation and the interaction with RNA splicing machinery, showing a positive correlation with exon inclusion in the mouse testis and brain.

### 4.2. H3ac and H4ac

Direct evidence supported the hypothesis that local histone acetylation surrounding alternative exons influences splice site selection. Histone hyperacetylation leads to an increased local elongation rate of RNAPII and decreased inclusion of exons [34,40,41]. For instance, a depolarization of human neuronal cells produces a hyper-acetylation of H3K9 (H3K9ac) in proximity to the alternatively spliced exon 18 of the *Ncam1* gene (neural cell adhesion molecule 1), resulting in increased exon skipping [41].

Two other examples of the role of histones acetylation in splicing include the neurofibromatosis type 1 (*Nf1*) tumor-suppressor gene, which contains alternative exon 23a [42], and the apoptosis-promoting receptor *Fas*, which generates a soluble isoform when alternative exon 6 is excluded [43]. In neuronal cells, the inhibition of HDAC2 deacetylation activity mediated by Hu proteins, which are splicing regulators, can lead to a higher histone hyperacetylation surrounding these two alternative exons, resulting in an increase in local transcriptional elongation rate and a reduction in exon inclusion [18,40].

### 4.3. H3K4me

In mammalian cells, the histone mark H3K4me3 was demonstrated to support effective splicing via the chromatin-splicing adaptor mechanism. H3K4me3 levels are important for early spliceosome binding to the human Cyclin D1 pre-mRNA through the chromatin-adaptor protein CHD1. Following that, CHD1 can recruit U2 small nuclear ribonucleoprotein (snRNP), through the SF3a complex [31]. Moreover, the high upstream H3K4me3 signal is stronger in alternatively spliced exons compared to constitutively spliced exons in lncRNA genes, where high-density regions of H3K4me3 correlate with intron retention [44].

Integrated analysis of ChIP-seq and RNA-seq data from 7 different mouse embryonic tissues at 6 developmental time points indicated that H3K4me1 is enriched in the regions downstream the 5′ splice site and upstream the 3′ splice site of skipped exons and that it is associated with exon inclusion [45].

### 4.4. H3K9me

The dimethylation (H3K9me2) and trimethylation (H3K9me3) of histone H3 lysine 9 correlate with transcriptional inhibition. Interestingly, recent data suggest that the H3K9me3 mark may have an effect on AS.

Local H3K9me3 changes can affect AS by labeling the chromosomal areas flanking alternative exons, and can have a considerable effect on exon inclusion [46,47]. The chromodomain protein HP1γ, which is generally classified as a transcriptional repressor, recognizes H3K9me3 marks in this area and allows incorporation of alternative exons via a process involving reduced RNAPII elongation rate. According to this study, the enrichment of H3K9me3 in the region comprising the variable exons of the gene encoding for the cell surface adhesion receptor CD44 corresponds with greater inclusion of the alternative exons in the mature mRNA. Similarly, the enrichment in H3K9me3 and HP1γ favors inclusion of variant exons of the genes encoding PKN2 (serine/threonine-protein kinase N2) and TAF4B (TATA-box binding protein associated factor 4).

As for H3K9me2, it was found associated with alternative exons in the Fibronectin (*Fn1*) gene that has a key role in growth and differentiation, as well as in cell adhesion and migration. Local H3K9me2 marks and the consequent binding of heterochromatin protein HP1α slows down the transcriptional elongation rate and allows the inclusion of exon E33/EDI [18,48]. 

### 4.5. H3K36me3

The trimethylation of histone H3 lysine 36 (H3K36me3) is a hallmark of transcription elongation. As a result, H3K36me3 levels are lowest near gene promoters and highest in transcribed regions. In addition, nucleosomes preferentially located in exons are enriched in the H3K36me3 histone mark [21]. In particular, H3K36me3-containing nucleosomes are abundant along intron–exon borders, and the enrichment level of this mark at alternatively spliced exons has been shown to correlate with their inclusion in the spliced transcript [29,47,49]. Moreover, the genome-wide correlation of histone PTMs and AS regulation during mammalian tissue development showed that H3K36me3 is highly informative of skipping-exon inclusion [45].

H3K36me3 can establish a chromatin platform for the splicing regulator polypyrimidine tract-binding protein (PTB) in the nascent pre-mRNA, via the adaptor protein MRG15 [28,50]. This H3K36me3-MRG15-PTB complex forms a chromatin-splicing adaptor system regulating numerous splicing events, including the splicing of the fibroblast growth factor receptor (*Fgfr2*). *Fgfr2*, which has two mutually exclusive exons (IIIb and IIIc), is a well-documented example of how H3K36me3 affects AS of a mammalian transcript. The *Fgfr2*-IIIb splice variant is expressed in epithelial cells, while *Fgfr2*-IIIc is expressed in mesenchymal cells. Luco and colleagues reported that local deposition of H3K36me3 can inhibit exon IIIb inclusion in mesenchymal cells [28].

Additionally, the short isoform of the chromatin-associated protein PSIP1 (PC4 and SFRS1 Interacting Protein 1) can identify the H3K36me3 mark and consequently control AS [27]. Indeed, the distribution of PSIP1/p52, which is enriched in expressed genes, correlates with H3K36me3 marks and can regulate AS through the recruitment of the splicing factor SRSF1 (Serine/Arginine-Rich Splicing Factor 1) to pre-mRNA.

### 4.6. Combinatorial PTMs Code

The combined impact of histone alterations on AS has also been described, both at the single-gene and genome-wide levels.

In a developmental setting, H3K36me3, H3K27ac, and H4K8ac were found to be differently enriched in half of the alternatively spliced events that change during human embryonic stem cell differentiation [51]. Shindo et al. [29] showed that H3K36me3, H3K4me3, H2BK12ac, and H4K5ac cooperate to regulate AS in IMR90 lung fibroblasts, analyzing the *Bin1* gene as an example. 

As described above, the inclusion of exon 33 (E33/EDI) in the *Fn1* gene has been associated with a more compact chromatin conformation, which is not only induced by the di-methylation of H3K9 but also by the concurrent tri-methylation of H3K27 in close proximity to the alternative exon [48]. Observations on the *Fgfr2* gene provided additional evidence for combinatorial histone-mediated alternative splicing control. In mesenchymal cells, the expression of the tissue-specific *Fgfr2* alternatively spliced isoform, *Fgfr2*-IIIc, is associated with the enrichment in H3K36me3 and H3K4me1, while in epithelial cells, the gene is enriched in H3K27me3 and H3K4me3 and is spliced into the *Fgfr2*-IIIb isoform. Notably, modulating H3K36me3 or H3K4me3 levels via overexpression or downregulation of their respective histone methyltransferases results in a predictable alteration in the tissue-specific alternative splicing pattern [28].

A genome-wide examination of 42 histone modifications revealed that they are not distributed randomly throughout the genome and that certain modifications are localized especially in exons relative to their adjacent intronic regions [17,20,52,53,54]. While the enrichment of many histone modifications is a result of the increased density of nucleosomes at exons, certain histone modifications, such as H3K36me3, H3K4me3, and H3K27me2, remain elevated after normalizing for nucleosome enrichment, while others, such as H3K9me3, are reduced.

In support of histone modifications serving as quantitative indicators of exon expression, four major groups of histone marks have been found in CD4+ T cells [53]. The first class (Class 1) includes histone modifications, such as H3K36me3, H3K79me1, and H2BK5me1, with a decreasing exonic signal from highly expressed to less expressed exons. Interestingly, an opposite tendency both at the exon- and gene-expression levels was observed for the second class (Class 2), which includes the H3K27me2 and H3K27me3 marks previously linked with gene silencing. The third class (Class 3) contains the active mark H3K27me1, which displayed a comparable signal in highly and moderately expressed exons but a weaker signal in lowly expressed exons. A fourth class of histone modifications (Class 4) was defined by the presence of H3R2me1 and H3K36me1 and was distinguished by a steady signal across all three exon expression levels.

More recently [55], the combinatorial changes of 38 different histone modifications in the exon skipping process of the same CD4+ T cells were further analyzed. The ChIP-seq data for 20 different types of histone methylation and 18 different types of histone acetylation were analyzed in three positions: excluded exons, included exons and the nearest 180 bps flanking intronic regions before and after the exons. The construction and analysis of probabilistic graphical models that represent a set of variables and their conditional dependencies (Bayesian networks) showed that some histone changes are strongly associated with RNA splicing. H3K79me3, H3K79me2, H3K4me2, H4K16ac, H3K4me1, H3R2me1, H4K5ac, H2BK120ac, H3K18ac, and H3K4ac are the ten histone modifications that have a direct association with RNA splicing in excluded exons. Conversely, the other 13 histone modifications (H3K79me1, H3K36me3, H3K36me1, H3K4me1, H3K4me2, H2BK12ac, H3K27ac, H2AK5ac, H4K16ac, H3K4ac, H412ac, H2BK120ac, and H3K18ac) show direct associations with RNA splicing in the included exon events. 

Additionally, a supervised machine learning approach was applied to large epigenomics datasets in human H1 embryonic stem cells and IMR90 fetal fibroblasts [26]. Based on exon inclusion levels, 11 chromatin changes differently identified 34% of all alternatively spliced exons in H1 human embryonic cells. Individually, there was no clear correlation between abundance of a certain histone mark and exon inclusion. However, when histone PTMs were analyzed in combination, seven distinct subsets of chromatin marks showing a position-dependent enrichment were discovered, resulting in splicing-associated chromatin signatures (SACS). Included exons are marked by H3K4me1 with H3K4me2 at exon boundaries (SACS1), H3K9me3 at exon boundaries (SACS2), and H4K20me1 with H4K91ac in the exon body (SACS3). In addition, excluded exons are characterized by H4K20me1 with H3K79me2 in the exon body (SACS4), H3K9me3 in the exon body (SACS5), H3K27me3 with H3K4me3 in the exon body (SACS6), or H3K9ac with H3K14ac upstream the exon (SACS7). Moreover, alternatively spliced exons were smaller than constitutive exons and each SACS group was enriched in different Gene Ontology biological processes, suggesting that chromatin may differently label exons that share common functional and/or regulatory pathways. 

The main findings related to the effect of histone modifications and variants on splicing outcome, which have been discussed above, are summarized in Table 1.

## 5. Histone PTMs and Altered Splicing Events in Cancer

**Figure 2:**
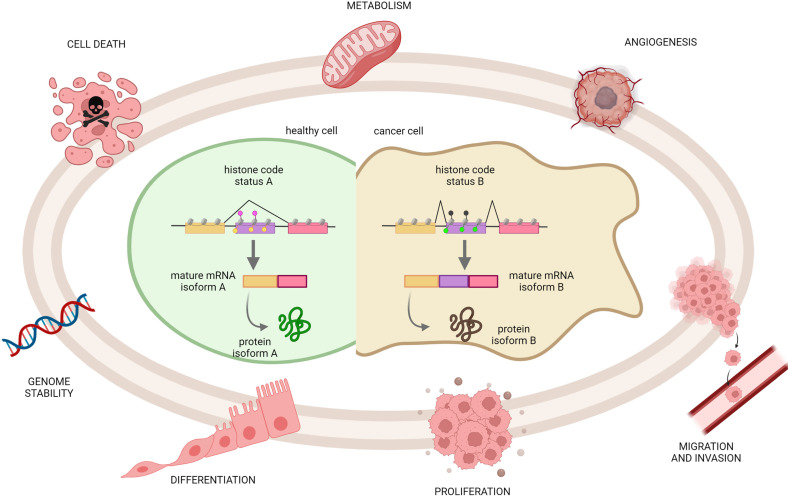
Genes undergoing alternative splicing produce functionally alternative products that can have dramatic effects on physiological processes and, consequently, can participate in the development or progression of cancer. The model represents how intragenic histone PTMs can drive distinct AS outcomes associated with common cancer hallmarks, such as genome instability, reduced cell death and differentiation, enhanced proliferation, metabolic reprogramming, and induced cell migration/invasion. Created with BioRender.com.

Different levels of histone marks have been clearly associated with the rate of gene transcription as well as splicing events. Cancer cells are characterized by misregulated splicing, which produces specific cancer transcripts and proteins involved in cancer initiation, maintenance, progression, and/or therapeutic resistance [56]. In particular, the aberrant splicing of gene transcripts significantly contributes to the transcriptional reprogramming of cancer-related pathways, including metabolism, cell cycle control, epithelial-to-mesenchymal transition (EMT), invasion, and metastasis. Moreover, AS can produce protein isoforms with opposite, even antagonistic, functional activities that severely impact on cancer biology [11] (Figure 2). 

Several mechanisms trigger aberrant splicing in cancer cells, such as somatic mutations in components of the human spliceosomal proteins and RNA splicing factors or point mutations near splice sites that promote gene mis-splicing [57,58]. In addition to altered splicing of specific genes, tumor cells show global splicing abnormalities induced by unknown triggers. Evidence of epigenetic alterations occurring in cancers suggests that aberrant AS on a genome-wide level can be the consequence of dysfunctional epigenetic splicing code, including altered histone PTMs enrichment [59]. 

Podlaha et al. found associations between relative isoform changes in expressed genes and enrichment levels of H3K4me1/2/3, H3K9ac, H3K9me3, H3K27ac, H3K27me3, H3K36me3, H3K79me2, and H4K20me in normal and cancer cell lines, and hypothesized that epigenetic regulation of transcript isoform diversity may be a common genome-wide phenomenon altered in tumor development [60]. Li et al. discovered a common pattern of histone marks, particularly H3K79me2, promoting exon inclusion in cell lines derived from hematological malignancies [61]. The integration of all available matched RNA-seq and H3K79me2 ChIP-seq data in the same cells suggested that a correlation exists between H3K79me2 and splicing events, through a co-transcriptional pre-RNA splicing mechanism independent of gene expression.

A recent study reported a strong correlation in time between dynamic changes in splicing of classical EMT genes and highly localized changes in H3K27me3, H3K27ac, and H3K4me1 levels in human epithelial MCF10a cells reprogrammed into mesenchymal-like cells [62]. Exon-specific regulated recruitment of chromatin effectors occurs to individual genes participating in EMT reprogramming, one of the main mechanisms through which cancer cells invade and metastasize. These changes in chromatin modifications are not only dynamic, as splicing events, but also reversible following MET (mesenchymal-to-epithelial transition). Moreover, Segelle et al. further demonstrated that H3K27 marks do not regulate splicing by modulating RNAPII elongation rates, but rather, they modulate the direct recruitment of specific splicing factors to the pre-mRNA.

In addition, in HPV+ oropharyngeal squamous cell carcinoma (OPSCC), a specific enrichment in H3K27ac has been identified as a key regulator of the expression of AS variants that contribute to OPSCC oncogenesis [63]. The treatment of HPV+ head and neck cell lines with JQ1, the inhibitor of the BRD4 (Bromodomain Containing 4) protein that recognizes the H3K27ac histone mark, is able to downregulate cancer-specific AS isoforms and eventually inhibits tumor cell proliferation. Hence, this study provides evidence for histone PTMs as targets for therapeutics that can restore the normal chromatin landscape.

The correlation between epigenetics and cancer AS transcripts is further supported by studies on specific genes. H3K27 acetylation and H3K9 methylation have been linked to AS of the human *Ar* gene, coding for the Androgen Receptor, which plays pivotal roles in prostate cancer (PC), especially castration-resistant prostate cancer (CRPC). In particular, the *Ar-v7* transcript includes a cryptic exon located in the intronic sequence between exons 3 and 4 and it encodes a constitutively active AR form highly expressed in CRPC and associated with poor patient prognosis [64]. In more detail, the AR-V7 protein lacks the C-terminal ligand-binding domain, thus acquiring ligand-independent transcriptional activity that has a functional role in driving resistance to second-generation androgen receptor signaling inhibitors (ARSi), such as abiraterone acetate and enzalutamide [65]. The histone demethylase JMJD1A (Jumonji domain containing 1A, also referred to as KDM3A), which regulates the levels of H3K9 methylation marks, has been correlated to the decrease in *Ar-v7* levels, but not of the full-length *Ar* transcript, in PC cells. 

Another example of gene-specific AS alteration is represented by the DNA mismatch repair *hMlh1* (MutL Homolog 1) gene. Several aberrant, shorter transcripts (such as delEx6–9, delEx9–10, delEx10, delEx11, delEx10–11, delEx16, and delEx17) of the *hMlh1* gene were observed in colon cancer and gastric cancer patients and cell lines and in a Lynch syndrome cohort [66,67,68,69], but their specific functional activities have not yet been clarified. The expression of deleted *hMlh1* transcripts has been described also in patients with acute lymphoblastic leukaemia (ALL), with the delEx9–10 splice variant having a dominant negative effect on the mismatch repair function of the wild type protein [70]. Low levels of histone acetylation (H4K16ac and H3ac) and methylation (H3K36me3) have been identified along relevant regions and specifically correlate with aberrant transcripts in the *hMlh1* exon 10–11 region. Increasing histone acetylation by the HDAC inhibitor Trichostatin A (TSA) reduces ratios of delEx11 and delEx10–11 transcripts versus normal transcript in gastric cancer cells. Additionally, RNA-mediated knockdown of the specific histone methyltransferase SETD2 (SET Domain Containing 2, Histone Lysine Methyltransferase) induces a shift in AS pattern in favor of *hMlh1* delEx11 and delEx10–11 transcripts.

With SETD2 being frequently mutated or deleted in various cancers, its association with AS highlights a new role in tumorigenesis. Yuan et al. investigated global changes in mRNA-splicing variants and identified more than 700 genes, many of which are involved in oncogenesis, in the intestine tissues of SETD2-deficient versus control mice [71]. ChIP-seq analysis showed a robust overlap between AS changes and genes with a reduction in the H3K36me3 mark in their bodies. A subset of these genes exhibited intragenic enrichment in RNAPII at AS sites, consistent with a decrease in RNAPII elongation. In particular, the role of SETD2 in intron retention was demonstrated in mRNA of the *Dvl2* gene that encodes for a member of the disheveled (Dsh) protein family, participating in the WNT pathway. The excision of intron 2 observed in SETD2-depleted cells can be ascribed to reduced H3K36me3 around the intron, with a consequent slow-down in RNAPII elongation, finally resulting in increased WNT signaling.

The impact of dysregulated SETD2 and, hence, H3K36me3 on AS transcripts has been further explored in renal cell carcinomas (RCC) [72]. SETD2 deficiency in RCC cells has been associated with the suppression of autophagy, as a consequence of the altered expression of AS isoforms of the *Atg12* gene, encoding for a component of the autophagy core machinery. Specifically, SETD2 depletion increased the expression of the inactive short ATG12 isoform, at the expense of the canonical active long isoform. 

Taken together, these data indicate that specific histone PTMs-targeting is a potential source of great opportunities for therapeutic tools that can modulate the expression of tumor splicing isoforms. 

## 6. Targeted Epigenome Editing: New Perspectives for Targeted Splicing Modulation

Recent research employing next-generation sequencing found genome-wide epigenetic and transcriptional correlations. Nevertheless, tools for direct targeted modification of epigenetic markers are required to convert association-based discoveries into mechanistic principles of gene regulation. As a result of the design and optimization of new tools, innovative platforms may become available for disease modeling, drug screening, gene therapy, and cell lineage characterization. 

**Figure 3:**
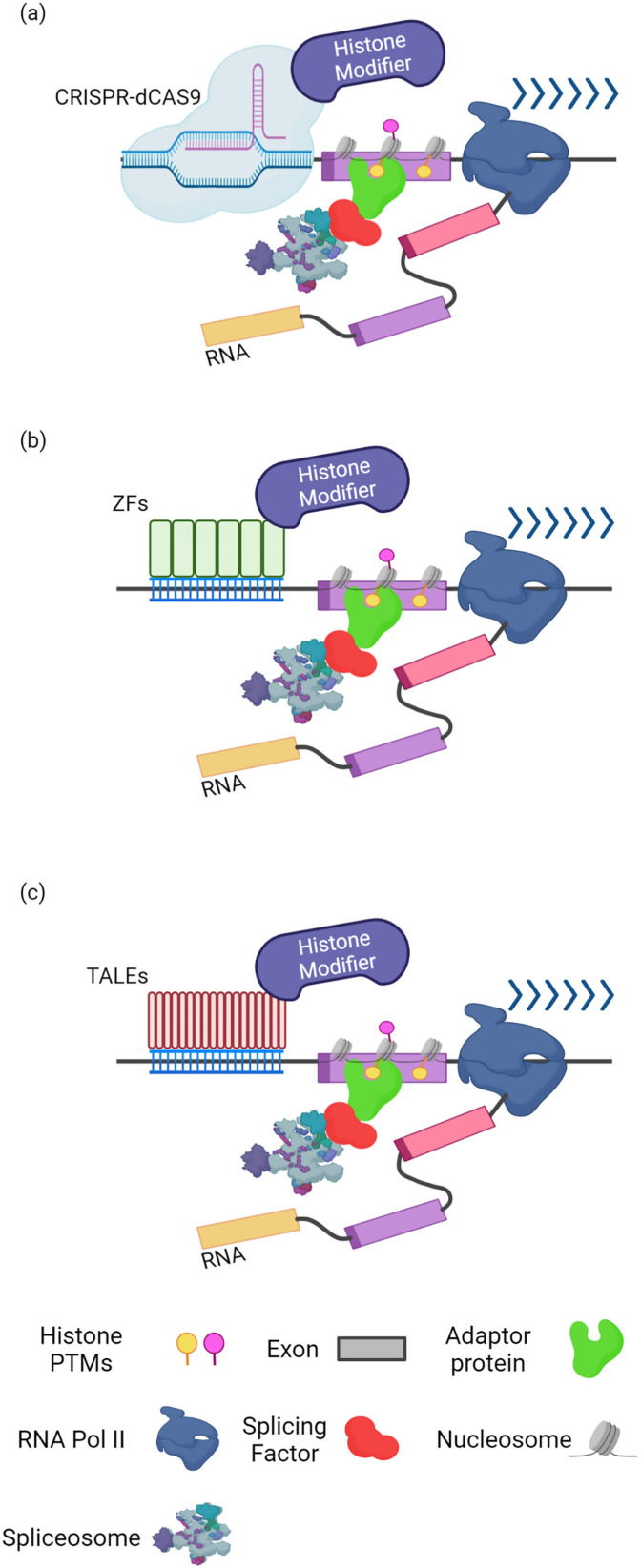
Overview of targeted epigenetic editing tools for localized modification of histone post-translational modifications. (**a**) CRISPR-dCas9 can target specific genomic locations by exploiting complementarity with a guide RNA. (**b**) Zinc Finger proteins can recognize 3 bps of DNA and the fusion of 6 ZFs can identify specific 18 bps sequences. (**c**) TALE domains can recognize a single-base pair and the fusion of several repeats distinguishes a specific locus. Created with BioRender.com.

Currently, chromatin function is studied using pharmacological and genetic perturbations, together with genome-wide assessment of gene expression and chromatin state. Even though these methods provided essential discoveries, they cannot directly evaluate chromatin regulatory function, due to global and pleiotropic effects. Furthermore, it is challenging to distinguish downstream from causal alterations. As discussed above, several findings showed that localized alterations in chromatin conformation and histone modification patterns along an alternatively spliced region can affect the result of splicing. Therefore, epigenetic modulation near splice sites can represent a therapeutic opportunity to rescue aberrant AS events (Figure 3).

Recent developments in programmable DNA-targeting technologies have emerged as a powerful tool to potentially generate epigenomic changes at any desired locus. Recent reports have shown that epigenome editing strategies based on the CRISPR-Cas9 system may directly modify particular modifications at specific genomic locations. However, the number of editable alterations and in vivo applications of these approaches remain limited. Exploiting complementarity between a synthetic guide RNA (gRNA) and the desired genomic sequence, the Cas9 nuclease can be directed to specific loci [73,74]. The Cas9 nuclease enzymatic activity can be abolished, resulting in a deactivated Cas9 (dCas9), and a programmable CRISPR-dCas9 system can localize effector domains to specific genomic sites, to manage epigenetic state (Figure 3a). A dCas9 linked to the catalytic domain of the human acetyltransferase p300 has been developed [73,75], revealing that this easily adaptable technique enables strong and highly selective control of the epigenome and subsequent gene expression. The Kruppel-associated box (KRAB) domain is a frequently used effector. When dCas9-KRAB fusions are targeted to DNA, they recruit a heterochromatin-forming complex that results in histone methylation and deacetylation [76]. Recently, Du et al. [77] created CRISPR Artificial Splicing Factors (CASFx) by fusing RNA-target Cas proteins with regulatory domains of the splicing factors RBFOX1 and RBM38. These CASFx could alter the inclusion of exon 7 of the endogenous survival motor neuron 2 gene (*Smn2*-E7) in GM03813 fibroblast cells derived from a type II spinal muscular atrophy (SMA) patient. To enable tunable control for CASFx, a rapamycin-inducible CASFx (iCASFx) was also generated. Direct modification of the epigenome was also obtained in *Medaka* (*Oryzias latipes*) embryos, where the repressive histone modification H3K27me3 was ectopically expressed in a locus-specific manner, using a fusion construct of the H3K27 methyltransferase Ezh2 (ol*Ezh2*) and dCas9 [78].

Aside from the CRISPR-Cas9 system, fusions of epigenome-modifying enzymes and programmable DNA-binding proteins, including zinc finger proteins (ZFs) and transcription activator-like effector (TALE) domains (Figure 3b,c), have been shown to be successful at changing chromatin in a targeted manner [79]. 

ZF nucleases were the first technology to be used for mammalian genome editing. Each zinc-finger can recognize 3 bps of DNA, and six adjacent zinc finger domains are often used to target 18 bps of DNA and confer specificity. ZFs were engineered to specifically target H3K9 methylation in endogenous genes [80]. Using ZFs coupled to a minimal catalytic domain of both SUV39H1 and G9A histone methyltransferases, ZFs were shown to alter local H3K9me in the endogenous *Vegf-a* promoter, thereby suppressing target gene expression. 

TALE effector repeats are DNA-binding domains that can bind virtually any genomic sequence [81,82]. TALE repeat domains linked to lysine-specific histone demethylase 1 (LSD1) were demonstrated to successfully eliminate enhancer-associated chromatin modifications from target loci, without altering control areas [83]. Moreover, in order to modulate H3K9 methylation in human cells, the TALE domain was fused to the H3K9 tri-methyltransferase SUV39H1, the catalytic domain of the di-methyltransferase G9a (EHMT2), or the H3K9me2/3 demethylase JMJD2D (KDM4D). The methyltransferases ASH1L and the catalytically active domain of SETD2 were also assessed for specific modulation of H3K36me3 [84]. The effect of alteration in H3K36 and H3K9 methylation was specifically investigated at exon 25 (EDII/EDB) of human fibronectin gene (*Fn1*) that was described to be sensitive to the chromatin environment [85,86]. While the effect of H3K36me3 modulation is apparently context-dependent and is not sufficient to alter the fate of the alternative target exon, the modulation of H3K9 methylation has a clear effect on AS of EDB-FN1.

## 7. Conclusions and Perspectives

Differential AS events have been associated with cancer biology and a relationship has been established between AS and the development of cancer features, such as an increase in proliferation, vascularization, and invasion. As a result, AS is now thought to be a novel hallmark of cancer. 

RNA-binding motifs are present in all cell types, indicating that they are not the main determinant of cell- and tissue-specific AS. Alterations in the expression of splicing factors cannot account for the detailed regulation of single exons, some of which must be included and others skipped, and others need the activation of cryptic splice sites, even within the same gene. Therefore, a histone-based system might encode information about AS patterns in different cell types and regions, in analogy to the histone indexing mechanisms used to specify which genes are expressed or silenced. This system may utilize histone changes to identify chromatin stretches encoding alternatively spliced areas, either to affect the rate of RNAPII elongation locally or to recruit splicing components via adaptor complexes. 

While global chromatin perturbations can teach us a lot, site-specific technologies allow us to look at chromatin regulation in a more physiological setting, without making severe alterations to the cellular environment. Antisense oligonucleotides (ASOs) have been thoroughly investigated in the therapeutic development process: targeted RNA treatment has been introduced in the clinic and ASOs may have therapeutic applications in cancer, according to preclinical studies showing encouraging outcomes with ASO-mediated modulation of splice variants in melanoma, pancreatic cancer, glioma, and breast cancer [87]. Moreover, recent breakthroughs in programmable DNA targeting technologies have opened up new possibilities for inducing epigenomic changes at any chosen locus. ZFs, TALE repeat domains, and the CRISPR/Cas system all offer distinct advantages and are all compatible with the approaches indicated above.

By shedding light on the mechanisms that connect histone PTMs and AS modifications, we may be able to make significant progress in understanding the molecular genetics of cancer cells and, as a result, in exploiting modern DNA targeting technology as therapeutic strategies to revert the expression of tumor splicing isoforms.

## Figures and Tables

**Table 1:** An Integrated Model for the Regulation of Alternative Splicing by histone modifications.

Histone Chromatin Marker	Influence on AS	References
Linker Histone variant H1.5	Exon inclusion	[35]
Histone variant H2A.Z	Exon inclusion	[36,37]
Histone variant H2A.B/H2A.B.3	Exon inclusion	[38,39]
H3K9ac	Exon exclusion	[40,41]
H3ac and H4ac	Exon exclusion	[41,42,43]
H3K4me3	Exon inclusion	[31,44]
H3K4me1	Exon inclusion	[45]
H3K9me3	Exon inclusion	[46,47]
H3K9me2	Exon inclusion	[18,48]
H3K36me3	Exon inclusion	[27,28,29,45,47,49,50]
H3K9me2 + H3K27me3	Exon inclusion	[48]
H3K36me3, H3K79me1, and H2BK5me1	Exon inclusion	[53]
H3K27me2 and H3K27me3	Exon exclusion	
H3K27me1	Exon inclusion	
H3R2me1 and H3K36me1	Exon inclusion	
H3K79me3, H3K79me2, H3K4me2, H4K16ac, H3K4me1, H3R2me1, H4K5ac, H2BK120ac, H3K18ac, and H3K4ac	Exon exclusion	[55]
H3K79me1, H3K36me3, H3K36me1, H3K4me1, H3K4me2, H2BK12ac, H3K27ac, H2AK5ac, H4K16ac, H3K4ac, H412ac, H2BK120ac, and H3K18ac	Exon inclusion	
H3K4me1 + H3K4me2 at exon boundaries (SACS1), H3K9me3 at exon boundaries (SACS2), H4K20me1 + H4K91ac in exon body (SACS3)	Exon inclusion	[30]
H4K20me1 + H3K79me2 in exon body (SACS4), H3K9me3 in exon body (SACS5), H3K27me3 + H3K4me3 in exon body (SACS 6), H3K9ac + H3K14ac upstream the exon (SACS7)	Exon exclusion	

## Data Availability

Not applicable.
